# Community-Associated Methicillin-Resistant Staphylococcus aureus: Case Report of Acute Sinusitis With Orbital Extension in a Pregnant Lady

**DOI:** 10.7759/cureus.12054

**Published:** 2020-12-13

**Authors:** Shazma Khan, Sarwar Siddiqui

**Affiliations:** 1 Neurology, Aga Khan University, Karachi, PAK; 2 Neurology, Ziauddin University, Karachi, PAK

**Keywords:** methicillin-resistant staphylococcus aureus, mrsa, sinusitis, headache

## Abstract

Methicillin-resistant Staphylococcus aureus (MRSA) infections pose a significant burden on healthcare. Acute sinusitis can be one of its deadliest presentations as it can quickly lead to orbitocranial extension with complications including blindness, brain abscess and death. Previously believed to affect immunocompromised individuals only, community-associated MRSA is now known to affect healthy individuals too. The seriousness of MRSA infection increases manifold when the infection occurs in pregnant women who are immune modulated. We present a case of a 34-year-old pregnant lady who presented with a severe headache for two days. She had acute sinusitis that involved right orbit in less than 24 hours. She was promptly managed with intravenous antibiotics and drainage of abscess that revealed MRSA. Fortunately, the patient made a complete recovery. The purpose of this case report is to emphasize on keeping a high index of suspicion of MRSA for all soft tissue infections. Early recognition, proper evaluation and timely and appropriate treatment can prove lifesaving.

## Introduction

Recognized since the 1960s, methicillin-resistant Staphylococcus aureus (MRSA) is well known to cause skin and soft tissue infections (SSTIs) and chronic sinusitis in immune-suppressed individuals and is generally considered hospital-acquired. A different strain, community-associated MRSA (CA-MRSA), is now believed to infect immune-competent individuals and pregnant women who undergo natural immune modulation during pregnancy and the postpartum period [1-3]. Some of the suspected risk factors for CA-MRSA include crowded living, poor hygiene and intravenous drug abuse [1]. In women, it tends to colonize the perineum and rectum due to frequent shaving, waxing and sharing hygiene products. CA-MRSA can lead to life-threatening infections in healthy pregnant women by a highly virulent exotoxin known as Panton-Valentine leukocidin (PVL) [1]. The risk of vertical transmission from mother to neonate can be significant [4,5]. Fortunately, the rate of maternal MRSA colonization is only about 0.5-4% [2]. It is still of vital importance to consider MRSA in all SSTIs as mortality rates are high in invasive infections [1,6].

## Case presentation

**Figure 1:**
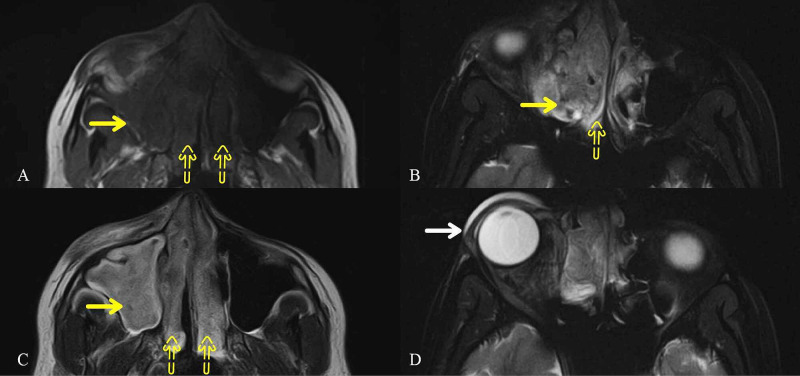
Orbital Cuts of MRI Brain almost complete opacification involving the right maxillary (solid yellow arrows) and bilateral ethmoid (yellow dotted arrows) sinuses, appearing (A) hypointense on T1 and (B, C) hyperintense on T2-weighted images. (D) There is mass effect causing slight proptosis of the right eyeball with preorbital soft tissue swelling (solid white arrow). MRI: magnetic resonance imaging

A 34-year-old-lady, known to have a migraine, presented to the emergency at 31 weeks gestation with a severe right-sided headache for two days. There was preceding continuous, mild headache for two weeks along with photophobia and phonophobia. The headache was partially responsive to paracetamol. She initially took it as her usual migraine, but her pain became severe, mainly involving the forehead and right periorbital region. There was no associated nausea, vomiting, fever or visual complaints. A thorough neurological exam was normal on the day of admission. The patient refused an MRI brain on day one, believing it to be harmful to the fetus. She was kept under observation and prescribed analgesics with an impression of migraine. A day later, her examination revealed partial right ptosis and right sixth nerve palsy with bilaterally reactive pupils and normal fundi. Serum leukocyte count and C-reactive protein were mildly raised. With differential diagnoses of ophthalmoplegic migraine, cerebral venous sinus thrombosis (CVST) or retro-orbital infection, MRI brain without contrast with orbital cuts and MR venogram (MRV) were obtained after counselling the patient. MRI brain revealed almost complete opacification of right frontal, maxillary and bilateral ethmoid sinuses with proptosis of the right eyeball and preorbital soft tissue swelling (Figure 1).

There was no evidence of cerebral venous sinus thrombosis. Infectious disease specialist was consulted immediately, and intravenous amphotericin was started with a high suspicion of fungal infection while bacterial coverage was started empirically with meropenem and vancomycin. Amphotericin had to be discontinued after the first dose due to an allergic reaction. Otorhinolaryngology consultation was solicited on day two when she developed proptosis of the right eye along with inflammation of the eyelid and periorbital region. An impression of acute bacterial sinusitis with cellulitis was made. The patient refused for sinus washout. Hence nasal endoscopy was done, and cultures were taken. On day three, the patient’s proptosis and headache worsened. Until then, three consecutive nasal swabs were negative for fungal or bacterial growth, and ongoing blood cultures were also negative. Late at night on day three, the patient had to be rushed to the operating room due to acute development of right-sided facial swelling and an unbearable headache. Right maxillary sinus washout was done that revealed frank pus. Meropenem and vancomycin were continued. Gram stain of pus drained from right maxillary sinus grew gram-positive cocci initially and revealed MRSA finally. Fetal health was unaffected by sinus drainage. The patient improved dramatically after the drainage of pus. Her headache was relieved, and proptosis and ptosis improved. On day five, she was discharged home with advice to continue intravenous antibiotics. On a follow-up one week after discharge, the patient had complete recovery without any complications.

## Discussion

Methicillin-resistant Staphylococcus aureus (MRSA) frequently colonizes the nostrils, perineum and skin breaks [1]. More frequently known to cause chronic rhinosinusitis, MRSA can also lead to full-blown acute invasive sinusitis [6,7]. Our patient developed extensive signs of orbital cellulitis leading to a subperiosteal abscess in less than a day. Hence any patient with a new headache or change in the character of headache should undergo a repeated examination to detect any change, which may warrant urgent medical or surgical attention [7]. Due to the unnecessary use of antibiotics, emerging resistance is a potential problem. Hence empiric antibiotic coverage was not provided in this case initially. However, as soon as the patient developed orbital cellulitis, broad-spectrum bacterial, as well as fungal coverage, was immediately commenced. Although fungi are considered the most typical cause of acute orbital infection, bacteria cannot be disregarded [1,8]. All seriously ill patients with SSTIs should be started on empiric coverage against MRSA based on local antibiogram [9]. The length of therapy should be 7 to 14 days but may vary based on the patient’s clinical condition [10]. Untreated sinusitis can lead to rapid orbital and intracranial extension, leading to grave consequences such as blindness, intracranial abscess, meningitis or death [7].

## Conclusions

MRSA infection should not only be considered in immunocompromised but also in immune-competent individuals because of the emergence of CA-MRSA strain. Although there is no apparent difference between MRSA and CA-MRSA, SSTIs among healthy individuals and immune modulated pregnant women have been attributed to the latter. Appropriate empiric antibiotics should be started without delay in all SSTIs and early drainage of the abscess, if any, should be the target. Delay in treatment, medical or surgical, can lead to severe consequences, including death.
